# Structural insights into acyl-ACP selective recognition by the *Aeromonas hydrophila* AHL synthase AhyI

**DOI:** 10.1186/s12866-021-02244-9

**Published:** 2021-06-08

**Authors:** Lei Jin, Jingjiao Bao, Yu Chen, Wenge Yang, Wenyi Du

**Affiliations:** 1 College of Food and Pharmaceutical Sciences, Ningbo University, Ningbo, 315211 China; 2 Key Laboratory of Animal Protein Food Deep Processing Technology of Zhejiang Province, Ningbo University, Ningbo, 315211 China; 3 Zhejiang Marine Fisheries Research Institute, Zhoushan, 316021 China; 4 Sichuan MoDe Technology Co., Ltd., Chengdu, 610000 China

**Keywords:** *Aeromonas hydrophila*, N-acyl-homoserine lactone, AhyI, Biosynthesis, Native acyl substrates, Selective recognition, Structural basis

## Abstract

**Background:**

*Aeromonas hydrophila* is a gram-negative bacterium and the major causative agent of the fish disease motile aeromonad septicemia (MAS). It uses N-acyl-homoserine lactone (AHL) quorum sensing signals to coordinate biofilm formation, motility, and virulence gene expression. The AHL signaling pathway is therefore considered to be a therapeutic target against pathogenic *A. hydrophila* infection. In *A. hydrophila*, AHL autoinducers biosynthesis are specifically catalyzed by an ACP-dependent AHL synthase AhyI using the precursors SAM and acyl-ACP. Our previously reported AhyI was heterologously expressed in *E. coli*, which showed the production characteristics of medium-long chain AHLs. This contradicted the prevailing understanding that AhyI was only a short-chain C_4_/C_6_-HSL synthase.

**Results:**

In this study, six linear acyl-ACP proteins with C-terminal his-tags were synthesized in *Vibrio harveyi* AasS using fatty acids and *E. coli* produced active holo-ACP proteins, and in vitro biosynthetic assays of six AHL molecules and kinetic studies of recombinant AhyI with a panel of four linear acyl-ACPs were performed. UPLC-MS/MS analyses indicated that AhyI can synthesize short-, medium- and long-chain AHLs from SAM and corresponding linear acyl-ACP substrates. Kinetic parameters measured using a DCPIP colorimetric assay, showed that there was a notable decrease in catalytic efficiency with acyl-chain lengths above C6, and hyperbolic or sigmoidal responses in rate curves were observed for varying acyl-donor substrates. Primary sequence alignment of the six representative AHL synthases offers insights into the structural basis for their specific acyl substrate preference. To further understand the acyl chain length preference of AhyI for linear acyl-ACP, we performed a structural comparison of three ACP-dependent LuxI homologs (TofI, BmaI1 and AhyI) and identified three key hydrophobic residues (I67, F125 and L157) which confer AhyI to selectively recognize native C_4_/C_6_-ACP substrates. These predictions were further supported by a computational Ala mutation assay.

**Conclusions:**

In this study, we have redefined AhyI as a multiple short- to long-chain AHL synthase which uses C_4_/C_6_-ACP as native acyl substrates and longer acyl-ACPs (C8 ~ C14) as non-native ones. We also theorized that the key residues in AhyI would likely drive acyl-ACP selective recognition.

**Supplementary Information:**

The online version contains supplementary material available at 10.1186/s12866-021-02244-9.

## Background

*Aeromonas hydrophila* is an opportunistic pathogen and ubiquitous inhabitant of various aquatic environments across the world. It infects fish, reptiles, amphibians, and mammals (including humans) [1, 2], and causes motile aeromonad septicemia (MAS), the most important bacterial disease in fish, frequent outbreaks of which lead to huge economic losses [3, 4]. Antibiotics are usually the first choice for prevention and treatment of *A. hydrophila* infections, but extensive antibiotic use leads to the development of multidrug resistance [5].

Many Gram-negative bacteria use autoinducers as signal molecules to alter expression of specific genes and enable population density control, a process termed quorum sensing (QS) [6, 7]. N-acyl-homoserine lactones (AHLs) are the best characterized QS signals and wide distributed in most gram negative bacteria [8]. AHL molecules possess a conservative homoserine lactone ring (HSL) and vary in acyl chain length from C_4_ to C_18_ and in backbone branching or unsaturation and decoration (i.e., 3-oxo or 3-OH substitution at the β-carbon) [9]. AHL-mediated QS has been implicated as an important factor in the virulence of some bacterial pathogens [10], for example, it enhances biofilm maturation, modulates the exoenzymes and hemolysin production, and is involved in regulating the type III and type VI secretion system in the zoonotic agent *A. hydrophila* [11–14]. Importantly, targeting AHL signaling circuit asserts less selective pressure for developing drug resistances, and small-molecule antagonists of AHL signaling are therefore an alternative to antibiotic usage to provide protection against the QS-dependent pathogenesis of *A. hydrophila* (Fig. S1).

The LuxI family of proteins are major AHL synthases which act as signal initiator proteins in the synthesis of specific N-acyl-homoserine lactones [15]. LuxI proteins utilize the precursors S-adenosyl-L-methionine (SAM) as the amino donor, and acyl-acyl carrier protein (acyl-ACP) / acyl-Coenzyme A (acyl-CoA) as the acyl donor [16–18]. Both acyl-ACP- and acyl-CoA-dependent AHL synthases undergo acylation and lactonization to synthesize AHL and release methylthioadenosine (MTA) via an acyl-SAM intermediate [19–21]. While SAM is a conserved substrate for all AHL synthases, AHL specificity is determined by the acyl chain of acyl-ACP or acyl-CoA substrates [22, 23].

The crystal structures of three LuxI members which are ACP-dependent AHL synthases have been resolved; *Pseudomonas aeruginosa* LasI (PDB Code: 1RO5) [24], *Pantoea ste*wartii EsaI (PDB Code: 1KZF) [21], and *Burkholderia glumae* TofI (PDB Code: 3P2F) [25]. Several co-crystal structures of various ligands in complex with BjaI (a CoA-dependent AHL synthase from *Bradyrhizobium japonicum*) were also recently released [19]. These LuxI-type AHL synthases all contained a similar α-β-α fold with a V-shaped cleft and two prominent active-site cavities, and were structurally similar to the GCN5-related N-acetyltransferases (GNATs) family [26]. The site-specific variants of LuxI suggest that some identified residues play integral roles in catalysis, and thus it is possible to establish the structural basis for substrate specificity. AHL synthase specificity is tight, but not absolute, and is likely affected by cognate acyl-ACP pool supply [27]. For example, a C_8_-HSL synthase *Burkholderia mallei* BmaI1 can utilize non-native acyl-ACP substrates from the *E. coli* acyl-ACP pool to synthesize nonspecific AHLs, although the catalytic efficiencies are lower than those when the native octanoyl-ACP (C_8_-ACP) is used [28]. While the general structural features that determine substrate selectivity in partial LuxI-type AHL synthase are clear, the details for others, such as AhyI, remain to be defined.

In *A. hydrophila*, AHL is typically produced by AhyI and recognized by AhyR receptors [29]. For a long time, AhyI was only regarded as a producer of short-chain AHLs (C_4_-HSL and C_6_-HSL) [30], however, our recent work has showed that it can synthesize six types of AHLs (C_4_-HSL, C_6_-HSL, C_8_-HSL, C_10_-HSL, C_12_-HSL, and C_14_-HSL) [31]. Because of the lack of in vitro biosynthetic assays for medium- and long-chain AHLs, there is no substantial evidence yet to determine whether the longer-chain AHLs observed were artifacts or not. In this study, we presented kinetic studies with multiple acyl-ACP substrates to verify the hypothesis that longer acyl-ACPs might be acyl substrates for AhyI. We also provide new insights into acyl-donor substrate preferences and the structural determinants of substrate specificity in AhyI.

## Results

### Synthesis of acyl-ACP substrates

ACP was overexpressed in *E. coli* carrying the plasmid pET28a-*acpP* and isolated primarily in the apo-form. Apo-ACP must first undergo 4′-phosphopantetheine (4′-PP) modification of the conserved Ser36 through a phosphodiester bond to form active holo-ACP, after which fatty acids can be bound via thioester linkage to the 4′-PP group thiol (Fig. S2) [32]. In this study, the *E. coli* strain carrying pET28a-*acpP* was co-transformed with pBAD-*acpS* (expressing the *E. coli* AcpS) enabling the transfer of 4′-PP from CoA to apo-ACP. The phosphopantetheinylation of apo-ACP was shown to be complete by urea-PAGE analysis (Fig. S3). The purified *Vibrio harveyi* acyl-ACP synthetase (AasS) was used to catalyze reaction of holo-ACP and free fatty acids which yielded the linear acyl chain of ACP substrates. These reaction products were also analyzed by urea-PAGE (Fig. S3) and no single holo-ACP bands on polyacrylamide gel were observed, indicating that each acyl-ACP biosynthesis reaction was complete. The hexahistidine (his6)-tagged ACPs had a higher molecular weight than the native forms, and while they were still active, their activity was somewhat lower than the native forms. However, for subsequent enzymatic analysis, the activity of his6-tagged acyl-ACPs was deemed to be sufficient.

### Analysis of acyl-ACP utilization pools

**Fig. 1:**
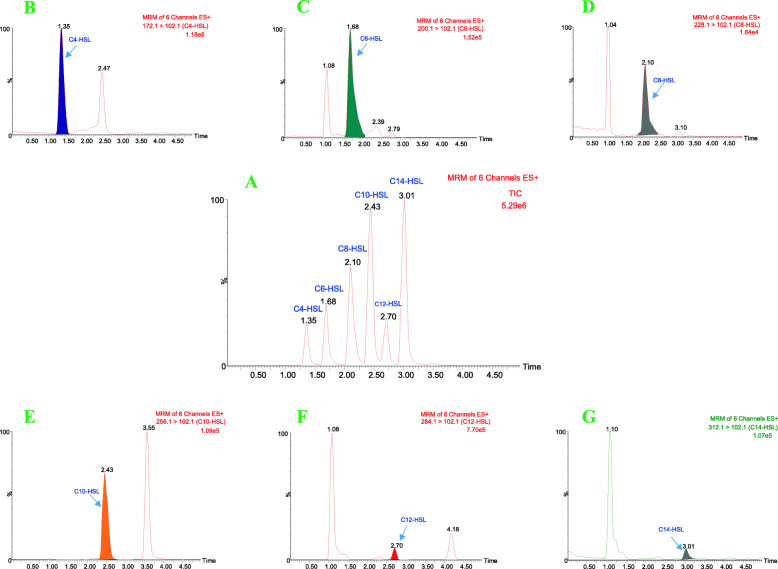
Typical UPLC-MS/MS ESI^+^ chromatograms from the AHL standard solutions (**A**) and in vitro enzyme reaction extracts, matching the retention times of all six AHLs, i.e., 1.35 min for C_4_-HSL (**B**), 1.68 min for C_6_-HSL (**C**), 2.10 min for C_8_-HSL (**D**), 2.43 min for C_10_-HSL (**E**), 2.70 min for C_12_-HSL (**F**) and 3.01 min for C_14_-HSL (**G**)

**Fig. 2:**
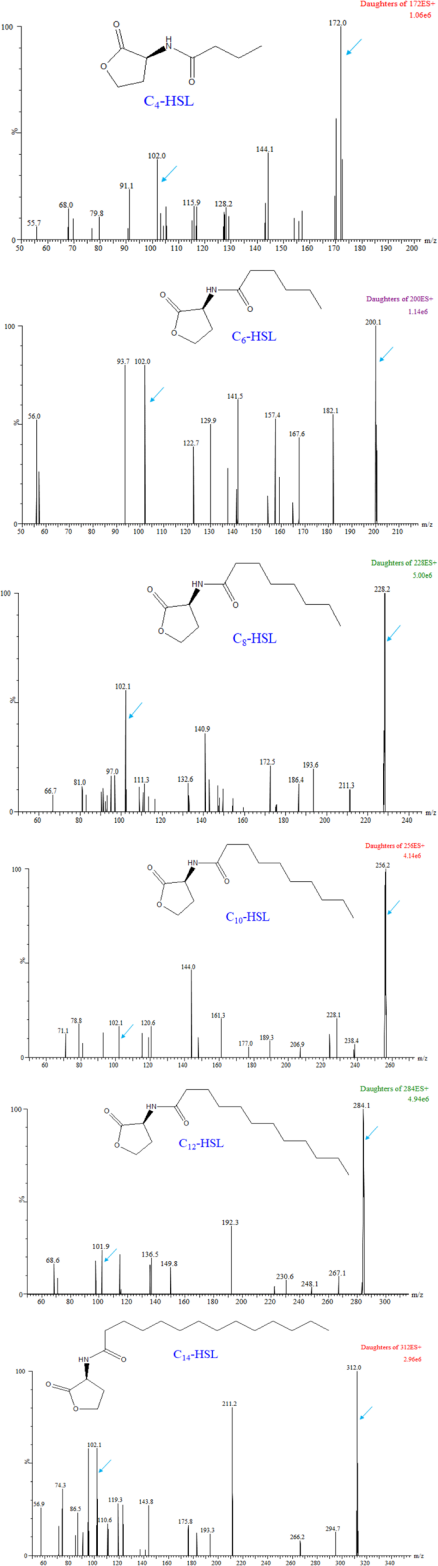
Mass spectra of six AHL products synthesized by AhyI via an in vitro enzymatic reaction. The characteristic peaks for respective C_4_-HSL (*m/z* 172.0 → 102.0), C_6_-HSL (*m/z* 200.1 → 102.0), C_8_-HSL (*m/z* 228.2 → 102.1), C_10_-HSL (*m/z* 256.2 → 102.1), C_12_-HSL (*m/z* 284.1 → 101.9) and C_14_-HSL (*m/z* 312.0 → 102.1) are marked by arrows

UPLC-MS/MS analysis of metabolites in cultured supernatants of recombinant *E. coli* carrying pET30a-*ahyI* confirmed production of six AHLs. To further verify AhyI’s in vitro enzymatic activity, we tested a substrate panel of linear acyl-ACP (C_4_ ~ C_14_-ACP) and SAM to observe formation of corresponding AHLs. Six typical characteristic peaks in total ion current (TIC) chromatograms were observed respectively by UPLC-MS/MS analysis, and were consistent with the retention times of the AHL standards (Fig. 1). All corresponding ion peaks for C_4_-HSL (*m/z* 172), C_6_-HSL (*m/z* 200), C_8_-HSL (*m/z* 228), C_10_-HSL (*m/z* 256), C_12_-HSL (*m/z* 284), and C_14_-HSL(*m/z* 312), and the precursor ion peak (*m/z* 102) matched those of the AHL synthetic standards in our previous experiments [31] (mass data shown in Fig. 2). These data suggest that AhyI can use all of these linear acyl substrates to synthesize the AHL products, including the short-chain C_4_/C_6_-HSLs and the medium-chain C_8_/C_10_-HSLs, as well as the long-chain C_12_/C_14_-HSLs. These in vitro experiments confirmed that acyl-ACPs with linear chains longer than C6 were also acyl-donor substrates for AhyI.

### Kinetics of AHL synthesis by AhyI

Kinetic analysis of AhyI against four linear acyl-ACPs (C_4_ ~ C_10_-ACP) was performed using a DCPIP colorimetric method, under fixed SAM conditions, and the kinetic parameters for AhyI using each of the acyl-ACPs as an acyl-donor substrate are described in Table 1. AhyI clearly favors C_4_-ACP with the lowest K_m_ (1.85 × 10^− 6^ M) and the highest *k*_cat_/K_m_ (12.29 × 10^3^ M^− 1^ s^− 1^) values. We saw that catalytic efficiency was severely affected as the acyl chain length increased. When compared to C_4_-ACP, the *k*_cat_/K_m_ values for C_6_-ACP, C_8_-ACP, and C_10_-ACP decreased more than 8-, 62-, and 175-fold, respectively, indicating that enzyme activity could be significantly inhibited when the acyl chain length is C8 or longer. These data agree with in vivo observations that more C_4_-HSL is found in the recombinant *E. coli* and *A. hydrophila* strains than any other AHL [31]. It should be noted that catalytic efficiencies were extremely low when the concentration of C_12_/C_14_-ACP substrates was less than 500 μM, and that there was great difficulty in preparing highly concentrated C_12_/C_14_-ACP proteins due to solubility issues with long-chain fatty acids (or salt) during the AasS reaction. We were unable to conduct kinetic studies for these two acyl substrates with AhyI when the fixed substrate was SAM.

**Table 1:** Kinetic constants for variable acyl-ACP substrates reacting with AhyI

Variable acyl-ACPs	*kcat* (s^−1^) × 10–3	Km (M) × 10–6	*kcat*/Km (M^− 1s − 1^) × 10–3	Relative ratioa
C4-ACP	22.73 ± 3.2	1.85 ± 0.16	12.29 ± 1.61	1
C6-ACP	19.44 ± 2.4	13.18 ± 1.9	1.48 ± 0.13	0.12
C8-ACP	8.41 ± 0.79	41.37 ± 8.9	0.20 ± 0.05	0.016
C10-ACP	6.34 ± 0.43	91.79 ± 15.5	0.07 ± 0.01	0.0057

## Discussion

In contrast to the *E. coli* DK574, DK574-pJT93 or DK574-pJT94 strains used in other studies to prepare holo-ACP [19, 28, 33], the *E. coli* holo-ACP expression system in this study was easily conducted and soluble holo-ACP with a hexahistidine tag could be routinely purified by Ni^2+^ affinity chromatography in most laboratories. Accumulation of unmodified apo-ACP will normally strongly inhibit *E. coli*. Growth [34], however, in our study, overproduction of *E. coli* ACP did not appeared to directly impact strain growth. This was attributed to the overexpression of holo-ACP synthase AcpS, which resulted in the rapid transformation of apo- to holo-ACP. In addition to the AaaS pathway, the phosphopantetheinyl transferase of Sfp from *Bacillus subtilis* (which will transfer the acyl-phosphopantetheine moiety of acyl-CoA to apo-ACP) is another commonly used approach for acyl-ACP synthesis [35]. However, compared to the two enzymatic methods, the acyl-ACP biosynthetic pathway in this study may be more economical due to the high price and incomplete commercial supply of acyl-CoA products.

**Fig. 3:**
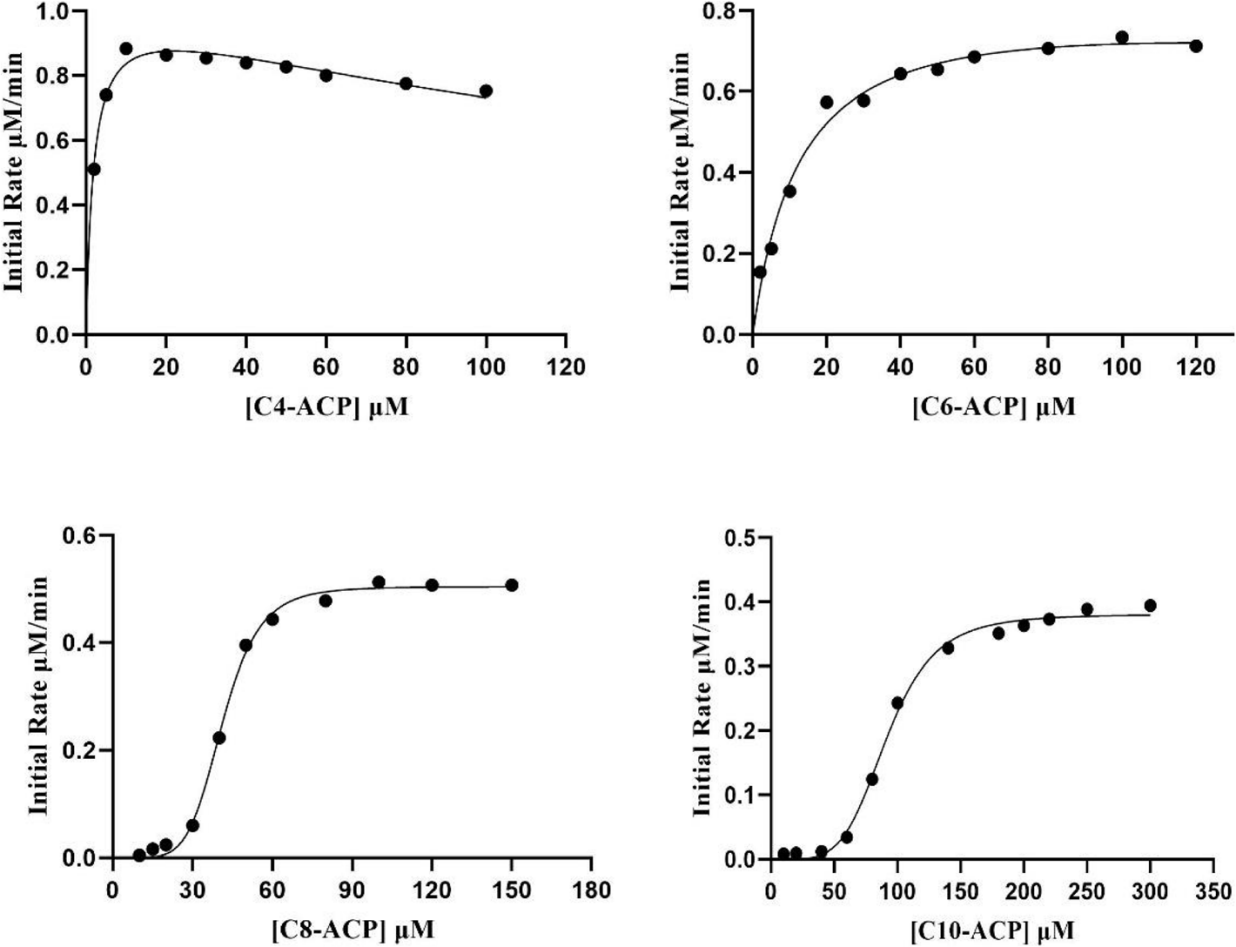
Substrate-velocity curves of AhyI with varying concentrations of acyl-ACPs. SAM was a fixed substrate at 1 mM, and enzyme concentrations were maintained at 0.75 μM (C_4_-ACP and C_6_-ACP) or 1 μM (C_8_-ACP and C_10_-ACP) in these experiments

Notably, the substrate-velocity curves were hyperbolic for C_4_/C_6_-ACP and sigmoidal for C_8_/C_10_-ACP (Fig. 3). Interestingly, substrate inhibition was seen in AhyI with C_4_-ACP utilization, which has also been observed for other LuxI type AHL synthases (e.g. BjaI and BmaI1) [19, 28]. Previous kinetic studies on BmaI1 established that hyperbolic behavior was appropriate for native acyl-ACP substrates with high reaction rates, and that a sigmoidal response in rate curves was seen with non-native acyl-ACPs [28]. Our kinetic data suggest that C_4_/C_6_-ACP are the native acyl-donor substrates for AhyI and others can be considered as non-native acyl-ACPs. It is therefore reasonable to assume that short-chain C_4_/C_6_-HSL are the specific (native) AHL products for AhyI and that nonspecific medium- and long-chain AHLs with low synthesis rates could disrupt intercellular communication. However, AHL-dependent regulation in *A. hydrophila* involving the medium- and long-chain AHLs has not been reported, and the effect (and impact) mechanism of these nonspecific AHL signaling molecules on QS regulation should be determined.

**Fig. 4:**
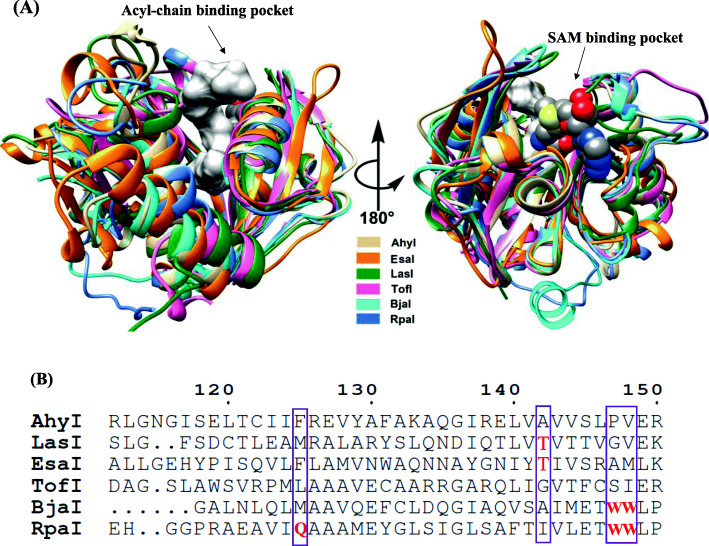
Common and unique structural characterization of six representative AHL signal synthases AhyI, EsaI, LasI, TofI, BjaI and RpaI. **A** Substrate-binding pocket showing the two cavities that accommodated acyl-donor and SAM. **B** Primary sequence alignment of these AHL synthases. Residues involved in the acyl-donor substrate preference were marked in red. The numbers indicate the AhyI residues

Our previous report of the AhyI model indicated the importance of a hydrophobic ligand pocket, hydrogen bonding interactions, and several crucial residues, with respect to AHL synthesis. However, the molecular detail of how AhyI selectively recognizes native acyl-ACP substrates from the cellular acyl-ACP pool has yet to be defined. As previously noted, nine hydrophobic residues (I67, L100, L103, F125, V144, I151, F152, L155, and L157) form an acyl-chain binding pocket in AhyI (Fig. S4), and in LuxI homologue proteins (EsaI, LasI, TofI, BjaI and RpaI) whose structures have been determined, similar hydrophobic residues have observed in a common deep-cavity that accommodates the hydrophobic acyl chain of the acyl-substrate (Fig. 4A). However, the acyl chain size and length of native acyl-donor substrates for these LuxI members varies. Sequence alignment of the six LuxI proteins, which represent the family of aryl-CoA- (RpaI), alkyl-CoA- (BjaI), linear alkyl-ACP- (TofI and AhyI) and 3-oxo alkyl-ACP- (EsaI and LasI) dependent AHL synthases (Fig. 4B), shows that AhyI lacks the canonical “indole platform”, resulting in a binding pocket that is more suitable for an acyl-ACP substrate, rather than an acyl-CoA one. Two Trp residues in BjaI (W142, W143) and RpaI (W146, W147) establish the “indole platform” which provides the basis for acyl-CoA preferences [19, 36]. Hydrogen-bond formation between the side chain of Q124 in RpaI and the hydroxyl group of *p*-coumarate is critical for *p*C-CoA binding, reflecting the difference in aryl- and alkyl-CoA substrate preference between RpaI and BjaI [36]. The C3 carbonyl of 3-oxo-acyl ACP participates in a critical hydrogen bond with the hydroxyl of a hydrophilic residue in EsaI (T140) [21] and LasI (T142) [24]. In AhyI, the corresponding residue (A142) loses contact with the 3-oxo-containing substrate, which accounts for its preference for a non β-carbon-oxidative acyl-ACP substrate.

**Fig. 5:**
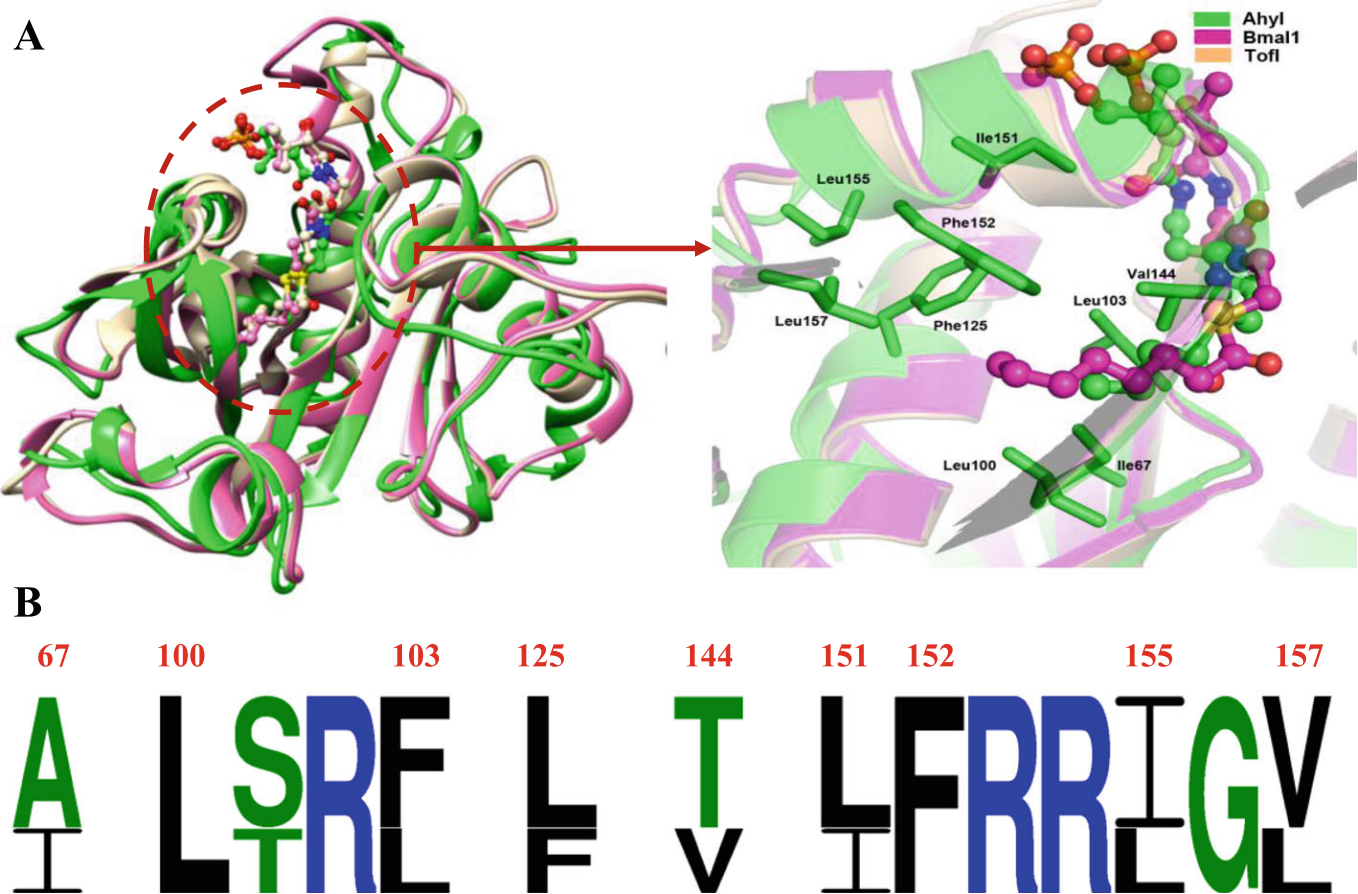
The structural alignment of TofI, BmaI1 and AhyI. **A** Overlapping ribbon diagram (left) of three linear AHL synthases in complex with respective native acyl substrates C8 (in pink) and C4 (in green). The closeup view (right) of acyl-chain binding pockets, with AhyI residues shown in sticks and colored in green. **B** Comparison of position-specific amino acid composition of acyl-chain binding sites based on WebLogo frequency plots, with the numbers indicating the AhyI residues

We focused next on AhyI’s acyl chain length preference for linear acyl-ACP, and made a structural comparison of linear AHL synthases TofI (C_8_-HSL), BmaI1 (C_8_-HSL) and AhyI (C_4_-HSL) (Fig. 5). A notable difference between the respective acyl-chain binding pockets is the replacement of small aliphatic residues (A68, L126 and V158) in TofI and BmaI1 with larger hydrophobic residues (I67, F125 and L157) in AhyI. This may mean that the binding pocket in AhyI can only accommodate shorter acyl substrates. Of these changes, the replacement of A68 with I67, which is three carbons longer, that with L100 are located at the bottom of the acyl-chain pocket in AhyI would likely restrict acyl chain length to C4 or C6. In AhyI, two key residues (L103 and V144) are located adjacent to the pocket periphery, but in TofI and BmaI1, larger residues (F105 and T145) are at the equivalent position, which could influence in ligand acyl chain selection. This may mean that an expanded set of longer acyl groups may be accommodated by AhyI. Residue variations at these positions provided a relatively reasonable explanation for how AhyI can utilize non-native acyl-ACP substrates (C8 ~ C14) with a limited acyl-chain pocket volume. A similar tunnel prediction had been recently verified by the observation of the increase in C_4_-HSL production and decrease in C_12_-HSL after a corresponding residue T105Y mutation in MplI [36].

**Fig. 6:**
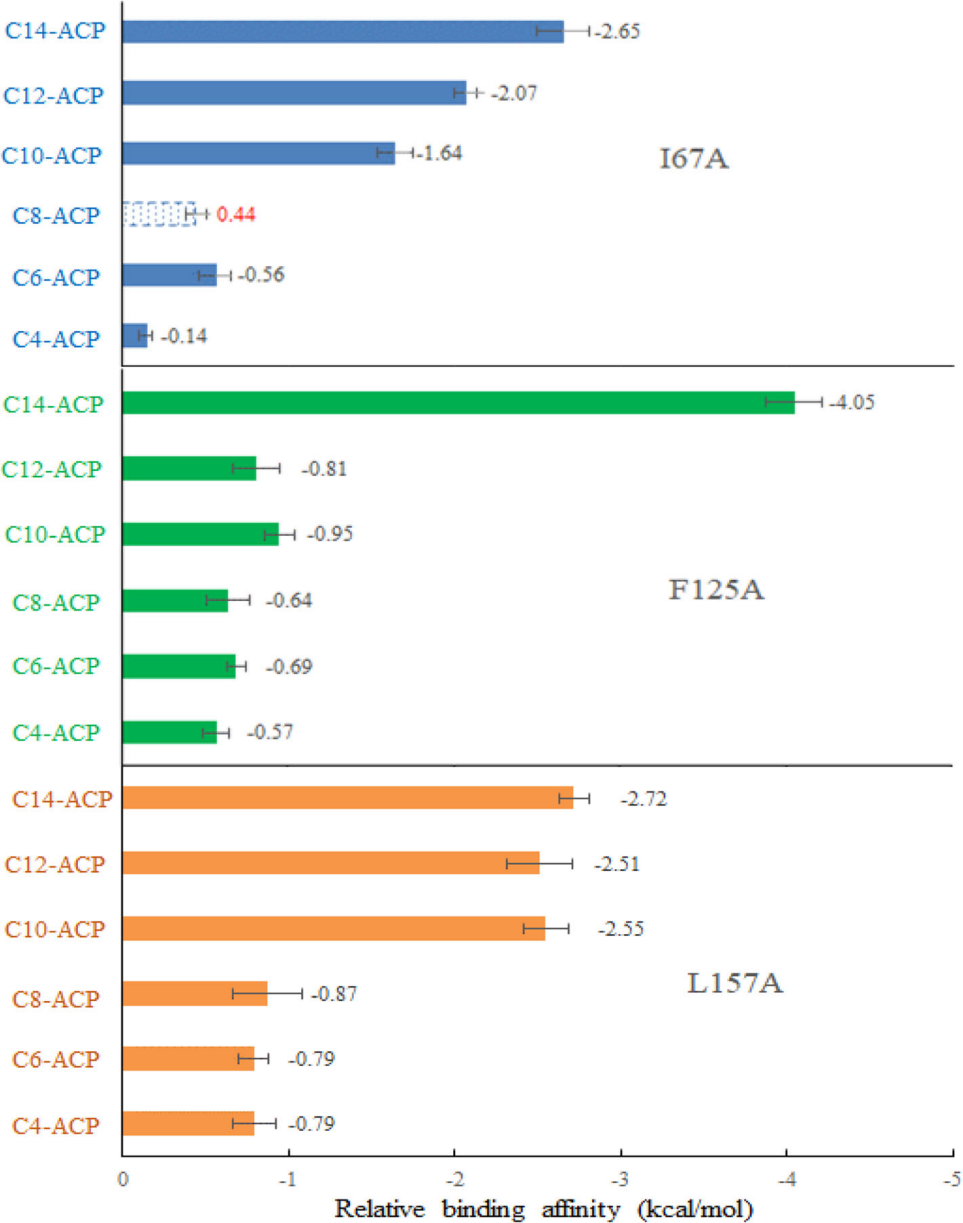
The relative (to WT) binding affinity obtained by docking acyl-4′-PP group of six acyl-ACP substrates into the binding pocket of wild-type (WT) AhyI or mutations. Blue dotted box with positive value colored red showed in the negative axis window, indicates the decrease in binding affinity

Dong et al. [19, 36] showed that the acyl-substrate tolerance of some CoA-based LuxI synthases is likely to depend on the volume of binding pocket, because residues important for acyl group binding were occuping the position that branched or linear alkyl-group of acyl-CoA substrates would be in. To further test the relationship between pocket size and acyl-ACP substrate tolerance, we carried out computational alanine mutation using AutoDock to compare the autodock-score values. Surface views of the acyl-chain pocket of WT AhyI with six acyl-ACP substrates is shown in Fig. S5. Auto dock results indicated that the relative binding affinities were increased upon introduction of mutations, with the exception of I67A which reduced the binding affinity to C_8_-ACP by 0.44 kcal/mol (Fig. 6). Notably, AhyI mutants had increased binding affinity for non-native acyl-ACPs, suggested that increasing the binding pocket volume would significantly facilitate AhyI recognition of medium-long chain acyl substrates. The computational data in the ligand binding models was insufficiency to prove the structure-function relationship, therefore, in future studies kinetic analysis of site-specific variants will be performed to better understand the mechanism of acyl substrate selective recognition in AhyI.

## Conclusions

Six AHL molecules were specifically produced by the ACP-dependent AHL synthase AhyI via in vitro enzymatic reaction, demonstrating that it is a multiple short- to long-chain AHL synthase. Kinetic studies with a panel of four linear acyl-ACPs suggest that C_4_/C_6_-ACP are AhyI’s native acyl-donor substrates and others with longer linear chains are non-native acyl-ACPs. A primary sequence alignment of six LuxI homologues indicated the non β-carbon-oxidative acyl-ACP substrate preference in AhyI is due to the lacks of an “indole platform” and the hydrogen bond interaction with the C3 carbonyl of a 3-oxo substrate. A structural comparison showed that there are three key hydrophobic residues (I67, F125 and L157) which are part of the acyl-chain binding pocket, and these were preliminarily proposed to be the structural determinants for native acyl-ACP selective recognition. Molecular docking simulations data further supports this proposition with the increased binding affinities for non-native acyl-ACPs seen in a representative subset of AhyI mutations. Our structural data are expected to provide theoretical direction for the molecular basis of native acyl-ACP specific recognition by AhyI.

## Methods

### Chemicals, plasmids and strains

AHL standards were purchased from Sigma-Aldrich Chemical Co. Chemicals for protein preparation and enzyme assays were purchased from Sangon Biotech (Shanghai) Co., Ltd., Bio-Rad Laboratories (Shanghai), Inc. or ProbeGene Inc. (Xuzhou, China). PCR primers and molecular biology reagents used for cloning vector construction were from Sangon Biotech. UPLC-MS/MS solvents and other conventional reagents were supplied by Merck KGaA (Germany) and Sinopharm Chemical Reagent Co., Ltd. (Shanghai, China).

AHL stock solutions (100 μM) were prepared with methanol. For holo-ACP purification, the following buffers were used; buffer A (20 mM Tris-HCl pH 8.0, 10 mM MgCl_2_, 5 mM DTT), buffer B (20 mM Tris-HCl pH 8.0, 2 M NaCl, 0.1% TritonX-100), buffer C (20 mM Tris-HCl pH 8.0, 50 mM NaCl, 0.1% TritonX-100) and buffer D (20 mM Tris-HCl pH 8.0, 5 mM DTT).

pET-His plasmids with *Escherichia coli* MG1655 ACP, *Vibrio harveyi* B392 AasS, and *A. hydrophila* HX-3 AhyI were from our previous work [31]. pBAD plasmid (no His-tag) with *E. coli* ACPs was a kind gift from Prof. Haihong Wang at the South China Agricultural University, Guangzhou. *E. coli* BL21(DE3) used for protein overexpression was obtained from in-house supplies.

### Purification of holo-ACP

pET28a-*acpP* and pBAD-*acpS* were co-transformed into *E. coli* BL21 (DE3) and positive clones were screened on LB medium containing kanamycin (50 μg/mL) and chloramphenicol (25 μg/mL) [37]. Bacteria carrying both plasmids was cultured at 37 °C with shaking in 2 L of LB media supplemented with 0.1 mM L-arabinose and the same antibiotics concentrations used above [38]. The culture was grown to an optical density of 0.8, and then induced with 0.5 mM IPTG for an additional 4 h. Cell pellets were harvested by centrifugation at 10000 rpm for 10 min at 4 °C and washed twice with equal volume of 20 mM Tris-HCl (pH 8.0). Cells were then resuspended in 10 mL ice-cold buffer A, and lysed by sonication. Cell debris was removed by centrifugation at 10000 rpm for 20 min at 4 °C, and 1 mM CoA was added to the supernatant and incubated for 4 h at 37 °C. An equal volume of ice-cold isopropanol was added to the extract and incubated with stirring at 4 °C for 1 h to remove most other proteins. Following centrifugation, the supernatant was concentrated under nitrogen to the half-volume and then dialyzed overnight against 50 mM MES, pH 6.1. The dialyzed extract was cleared by centrifugation and the supernatant was applied to a Ni-IDA column. The column was sequentially washed with 100 ml of buffer B, 20 ml of buffer C and 50 ml buffer C with 10 mM imidazole. Holo-ACP with a C-terminal his6-tag was eluted from the column using appropriate volumes of buffer C with 250 mM imidazole. The nickel ions and imidazole were removed by dialysis twice against buffer D. Purified holo-ACP proteins were concentrated to a volume of 4 mL and the final concentration was quantified by Nanodrop UV-Vis analysis using a molar extinction coefficient of 1.8 × 10^3^ at 280 nm [39]. The purity of the holo-ACP was monitored by using conformationally sensitive gel electrophoresis on a non-denaturing 17.5% polyacrylamide gel containing 2.5 M urea (urea-PAGE) according to previously used methods [33].

### Preparation of acyl-ACP substrates

*Vibrio harveyi* AasS was used to synthesize the linear acyl-ACPs (C_4_ ~ C_14_) [40]. A reaction mixture contained 100 mM Tris-HCl pH 7.8, 10 mM MgCl_2_, 5 mM DTT, 10 mM ATP, 100 μM fatty acid, 20 μM holo-ACP and 0.75 μM purified AasS and was incubated at 37 °C for 4 h. Additional reaction times (more than 12 h) were needed for the C_4_-ACP, C_12_-ACP and C_14_-ACP preparations. The reaction was stopped with the addition of 50% ice-cold isopropanol to remove AasS protein. The mixture was centrifuged and the suspension was treated with two volumes of acetone to precipitate acyl-ACP proteins and incubated at − 20 °C overnight [41]. Following centrifugation and two washes with three volumes of acetone, precipitates were air dried and resuspended in buffer D. Essentially complete conversion to acyl-ACPs was verified by urea-PAGE.

### In vitro assay of AhyI activity

The acyl substrate recognition profile of AhyI was analyzed using the reaction mixture (0.5 mL) containing 100 mM Tris-HCl pH 7.8, 1 mM SAM and 100 μM acyl-ACP (C_4_ ~ C_14_). Reactions were initiated by the addition of 1 μM purified AhyI obtained from our previous work [31], then incubated at 37 °C for 60 min. The AHL products in the reaction mixtures were extracted twice with an equal volume of ethyl acetate containing 0.01% glacial acetic acid. The organic phase was dried under nitrogen and residues were dissolved in 1.0 mL of methanol. The final products were validated by ultraperformance liquid chromatography-tandem mass spectrometry (UPLC-MS/MS) analysis using the method previously described [31].

To determine AhyI’s kinetic parameters, the enzymatic reaction was monitored using a colorimetric assay that measured the decrease in 2,6-dichlorophenolindophenol (DCPIP) absorbance at 600 nm [23, 42]. The typical reaction contained 30 μM DCPIP, 100 mM Tris-HCl pH 7.8, 1 mM SAM, and 2–300 μM acyl-ACP. After a 10 min incubation period, the reactions were initiated by addition of AhyI at 0.75 μM for C_4_-ACP and C_6_-ACP, or 1 μM for C_8_-ACP and C_10_-ACP. The reduction of DCPIP by free holo-ACP released in AHL synthesis was monitored at 600 nm (Δε_600_ = 21,000 M^− 1^ cm^− 1^) over 10 min and initial rates were calculated based on the progress curve. To estimate kinetic constants, the initial rate data were fitted to Michaelis-Menten or substrate inhibition equation using the Graphpad Prism 8.0.

### Molecular docking

The homology model of AhyI was generated with Modeler v9.19 and TofI structure (PDB code: 3P2F) used as a molecular template. The detailed method for model construction and refinement was described in our previous publication [31]. The initial structures of mutated AhyI (I67A, F125A and L157A) were prepared using PyMOL 1.8, following by energy optimization to allow the mutant structure to find the minimum energy conformation. The structures of acyl-4′-phosphopantetheine (C_4_-, C_6_-, C_8_-, C_10_-, C_12_- and C_14_–4′-PP) were processed with AutoDock Tools 1.5.6 by adding hydrogens and further optimized using the PM3 Hamiltonian in MOPAC. Molecular docking of acyl-4′-PP and AhyI was carried out using AutoDock 4.2.6 [43]. The grid box was set up with 60 × 50 × 60 points in the XYZ axes at a grid spacing of 0.375 Å. The number of Genetic Algorithm (GA) run was set to 100 and the default settings were used for the rest of the parameters. Finally, the optimal 3D docking conformations with lowest energy scoring were selected for computational Ala mutation assay. The binding affinity values from three parallel dockings of each protein-ligand were collected for further statistical analysis.

## Supplementary Information

**Additional file 1.**

## Data Availability

All data generated or analysed during this study are included in this published article and its supplementary information files. Protein sequences for EsaI, LasI, TofI, BjaI and RpaI can be obtained in PDB database under accession numbers: 1KZF, 1RO5, 3P2F, 5W8D and 6WN0, respectively.

## Abbreviations

AHL: N-acyl-homoserine lactone
SAM: S-adenosyl-L-methionine
ACP: Acyl carrier protein
acyl-ACP: Acyl-acyl carrier protein
C_4_-ACP: Butyryl-ACP
C_6_-ACP: Hexanoyl-ACP
C_8_-ACP: Octanoyl-ACP
C_10_-ACP: Decanoyl-ACP
C_12_-ACP: Dodecanoyl-ACP
C_14_-ACP: Tetradecanoyl-ACP
CoA: Coenzyme A
acyl-CoA: Acyl-coenzyme A
*p*C-CoA: *p*-coumarate-CoA
C_4_-HSL: N-butyryl-homoserine lactone
C_6_-HSL: N-hexanoyl-homoserine lactone
C_8_-HSL: N-octanoyl-homoserine lactone
C_10_-HSL: N-decanoyl-homoserine lactone
C_12_-HSL: N-dodecanoyl-homoserine lactone
C_14_-HSL: N-tetradecanoyl-homoserine lactone
QS: Quorum sensing
PAGE: Polyacrylamide gel electrophoresis
UPLC-MS/MS: Ultraperformance liquid chromatography-tandem mass spectrometry
IPTG: Isopropyl-β-D-thiogalactopyranoside
MES: 2-(N-morpholino) ethanesulfonic acid
