# Supplementary material for: Structural insights into acyl-ACP selective recognition by the Aeromonas hydrophila AHL synthase AhyI
Source: BMC Microbiol. 2021 Jun 8;21:173. doi: 10.1186/s12866-021-02244-9 (PMC8188788; doi:10.1186/s12866-021-02244-9)
Supplement: Supplementary file 1 — Additional file 1. [file 12866_2021_2244_MOESM1_ESM.docx]

**Supporting material**

**For**

**Structural insights into acyl-ACP selective recognition by the *Aeromonas hydrophila* AHL synthase AhyI**

**Contents**

**Fig. S1** Schematic diagram of biocontrol and prevention of MAS disease caused by *A. hydrophila* using AHL-QS target-specific agents.

**Fig. S2** The biosynthetic process of *E. coli* acyl-ACP in this study.

**Fig. S3** Urea-PAGE analysis of the various forms of ACP protein with C-terminal his6-tags.

**Fig. S4** The closeup view of acyl-chain binding pocket of AhyI in complex with acyl-4'-PP of C_4_-ACP and C_14_-ACP.

**Fig. S5** The optimal 3D docking conformation of acyl-4'-PP of six acyl-ACP substrates inserts into the binding pocket of WT AhyI protein.

Fig. S1 Schematic diagram of biocontrol and prevention of MAS disease caused by *A. hydrophila* using AHL-QS target-specific agents.


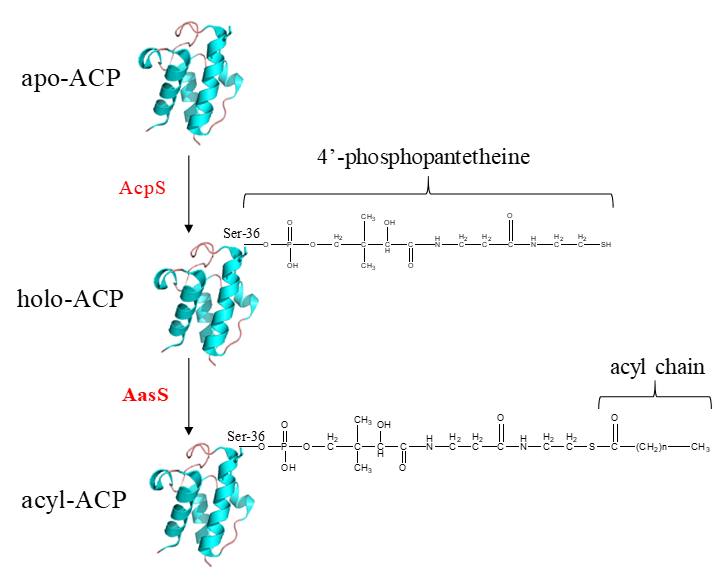


Fig. S2 The biosynthetic process of *E. coli* acyl-ACP in this study. The *E. coli* apo-ACP is modified with 4’-PP group in the position of Ser36 by AcpS reaction to form holo-ACP, and then acyl chain from fatty acid is attached as a thioester to 4’-PP group thiol using *V. harveyi* AasS.

Fig. S3 Urea-PAGE analysis of the various forms of ACP protein with C-terminal his6-tags. Fifteen μl of each ACP was run on 17.5% polyacrylamide gels containing 2.5 M urea. A mixture of apo- and holo-ACP standards was used as a marker (lane Stds). Purified holo-ACP was loaded in lane 1. The C_4_~C_14_-ACP products of the AasS reaction were shown in Lanes 2~7, respectively.


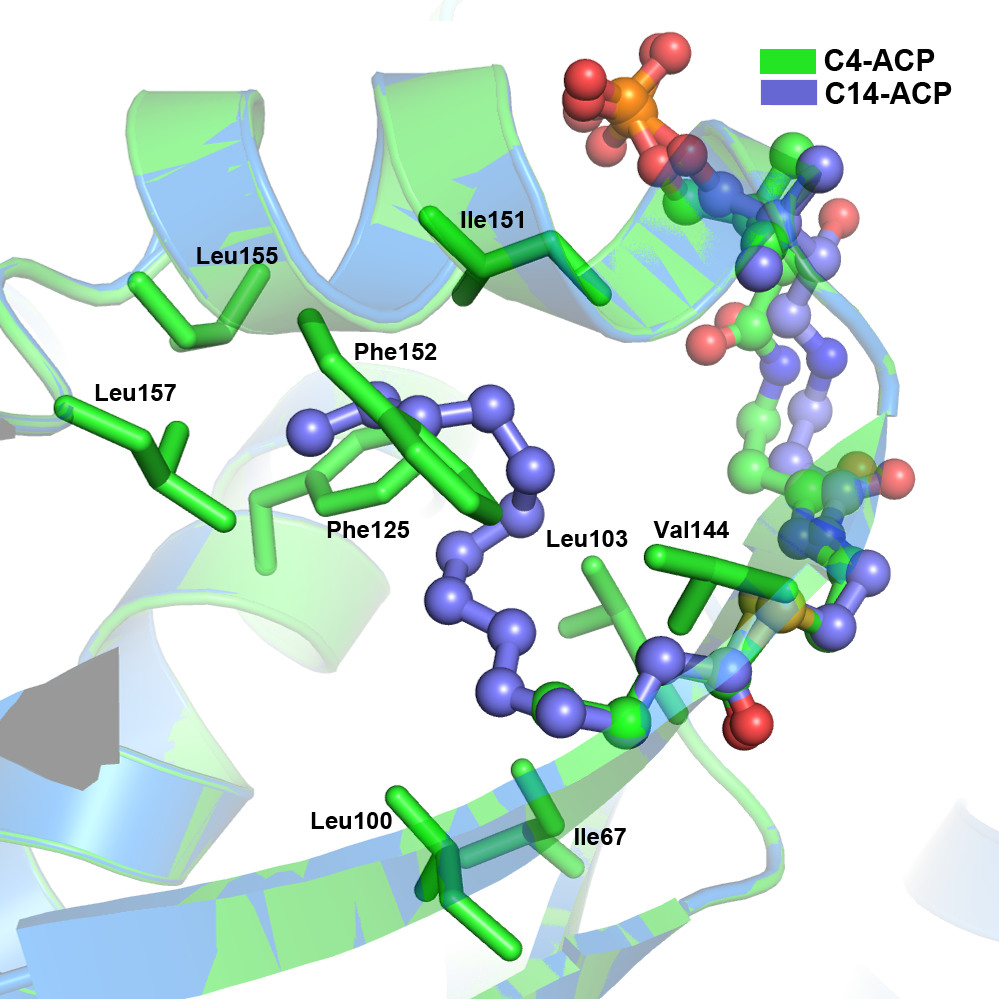


Fig. S4 The closeup view of acyl-chain binding pocket of AhyI in complex with a native short-chain C_4_-ACP (in green) and a non-native long-chain C_14_-ACP (in bluish violet). The AhyI residues were shown in sticks and colored in green.


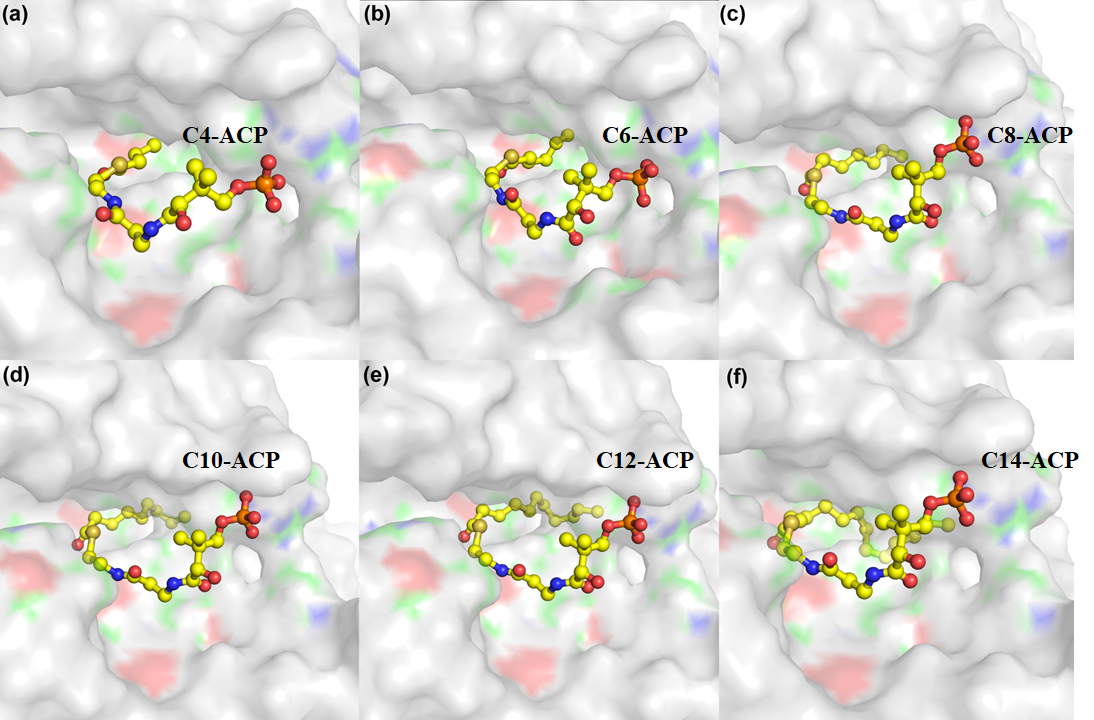


Fig. S5 The optimal 3D docking conformation of acyl-4'-PP of six acyl-ACP substrates inserts into the binding pocket of WT AhyI protein. Red, blue and green surfaces indicate the oxygen, nitrogen and carbon atoms of pocket residues surrounding the acyl-pantetheine group, respectively.
